# Fungal Endocarditis Secondary to Transdermal Fentanyl Patch

**DOI:** 10.7759/cureus.38706

**Published:** 2023-05-08

**Authors:** Bradley Casey, Amol Bahekar, Divyang Patel, Eric Walker, Amro Ilaiwy

**Affiliations:** 1 Internal Medicine, Cape Fear Valley Medical Center, Fayetteville, USA; 2 Cardiology, Cape Fear Valley Medical Center, Fayetteville, USA

**Keywords:** fentanyl patch, candidemia, trypanophobia, polysubstance abuse, fungal endocarditis

## Abstract

Fungal endocarditis is an uncommon and dangerous disorder of the heart. The two most frequent etiologic fungi discovered to be responsible for fungal endocarditis are Aspergillus and Candida species. It is difficult to make a diagnosis of fungal endocarditis; a comprehensive assessment must be carried out, and specific diagnostic requirements must be completed. One of the main causes of endocarditis that physicians deal with in the hospital is intravenous drug abuse, but we never hear about transdermal drug abuse causing endocarditis. Here we present an interesting case of a 33-year-old male patient that presents to the hospital with non-specific complaints, and he was found to have fungemia. It was found out that the patient was using a kitchen appliance to cause dermal abrasion on his skin to increase the absorption rate of his fentanyl patch. Patient also suffers from trypanophobia, so he declined any surgical intervention and wanted lifelong oral medication therapy.

## Introduction

As a result of an infection entering the bloodstream, traveling to the heart, and infiltrating coronary tissue, infectious endocarditis (IE) is mostly caused by *Staphylococcus aureus *[1]. The possible entrance points are dental work, urinary tract infections, lung infections, gastrointestinal infections, skin conditions, intravenous drug use, surgery, and intravenous cannulation from hospital admissions [2]. IE can also have the subclass of infectious fungal endocarditis (IFE), and this is a rare and fatal disease [3]. IFE is most commonly caused by Candida and Aspergillus species [3]. Most common causes of IFE are open-heart surgery, prosthetic grafts, central lines, long-term antibiotic therapy, intravenous drug abuse, preexisting congenital heart defects, immunosuppressed state, and prolonged corticosteroid use [4]. We present an interesting case of a patient that has trypanophobia (fear of needles) that presented to the emergency department for generalized weakness, fatigue, subjective fevers, and chills. The patient has been using a non-scratch scouring pad to cause inflammation on the surface of his skin, and then he would apply the fentanyl patch to cause an increased absorption rate. He was diagnosed with IFE and refused surgical intervention due to his trypanophobia. The patient was ultimately discharged home with long-term oral antifungal medication and had a full recovery.

## Case presentation

**Figure 1:**
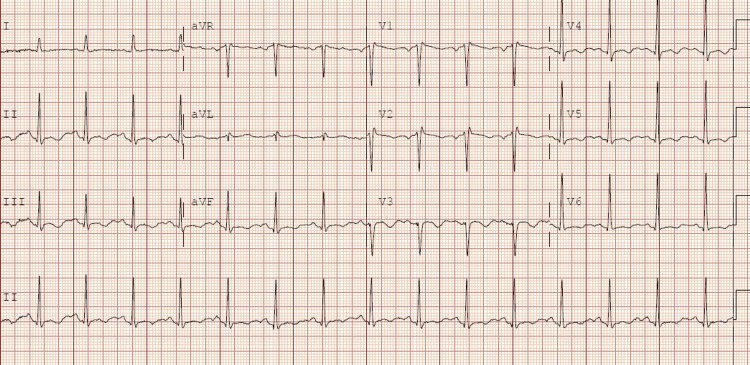
Electrocardiogram showed heart rate of 92 beats per minute with non-specific T-wave abnormalities.

A 33-year-old male patient with past medical history (PMH) of polysubstance abuse, trypanophobia, and hypertension presented to the emergency department with complaints of generalized weakness, fatigue, subjective fevers, and chills. The patient reported that he used cocaine and fentanyl patches daily. To increase the rate of absorption from his fentanyl patch he was using a non-scratch scouring pad to cause inflammation of the skin before applying the fentanyl patch. Upon evaluation in the emergency department, his vitals were significant for a temperature of 101.2° Fahrenheit and a heart rate of 103 beats per minute. During physical examination, the patient was found to have a new systolic murmur, which was not documented during previous hospitalizations. Initial electrocardiogram (EKG) can be seen in Figure 1, which showed sinus rhythm with a rate of 92 beats per minute and non-specific T-wave abnormalities.

Initial labs can be seen in Table 1. A complete physical examination of the skin showing multiple superficial abrasions, but was without any underlying fluctuance or significant overlying erythema. Due to the new systolic ejection murmur, a transthoracic echocardiogram (TTE) showed ejection fraction greater than 55%, trace mitral valve regurgitation, and thickening of the posterior leaflet of the mitral valve (Videos 1, 2). After the TTE was completed the patient’s blood cultures came back positive with *Candida albicans *(Table 1). Due to cultures returning positive and TTE showing thickened posterior mitral valve leaflet, a transesophageal echocardiogram (TEE) was performed. The TEE showed mobile mass on the posterior leaflet of the mitral valve that measured 8.5×4.5 mm with mild mitral regurgitation (Videos 3, 4). Cardiothoracic surgery was asked to come and evaluate the patient, and the patient declined any surgical intervention. Patient said that he was aware of the risk of being discharged without surgical intervention, but declined due to his trypanophobia. He said that he would rather do lifelong oral therapy as opposed to surgery. Since the patient had infectious disease, he was asked to come and be evaluated for most appropriate outpatient therapy, and antifungal susceptibility drug panel was completed (Table 1). Patient was discharged home on fluconazole with follow-up with primary care provider and infectious disease.

**Table 1:** Patient's laboratory values from hospitalization. MIC: minimum inhibitory concentration; AST: antifungal susceptibility testing

Variables	Laboratory test	Patient's laboratory test	Reference range
Complete blood count	White blood cell count	12.5×10^3^/uL	4.5-12.5×10^3^/uL
	Hemoglobin	13.5 g/dL	12.0-16.0 g/dL
	Mean corpuscular volume	95.2 fL	81.0-99.0 fL
	Platelets	310×10^3^/uL	150-450×10^3^/uL
Comprehensive metabolic panel	Sodium	134 mmol/L	136-145 mmol/L
	Potassium	2.9 mmol/L	3.5-5.1 mmol/L
	Bicarbonate	30 mmol/L	21-32 mmol/L
	Chloride	91 mmol/L	98-107 mmol/L
	Blood urea nitrogen	11 mg/dL	7-25 mg/dL
	Creatinine	0.70 mg/dL	0.60 mg/dL
	Glucose	115 mg/dL	74-106 mg/dL
	Aspartate aminotransferase	28 U/L	15-37 U/L
	Alanine transaminase	23 U/L	12-78 U/L
	Glomerular filtration rate	>60.0 mL/min/1.73 m^2^	>60.0 mL/min/1.73 m^2^
	Albumin	4.3 g/dL	3.5-5.7 g/dL
	Magnesium	2.5 mg/dL	1.9-2.7 mg/dL
Other blood tests	Ethanol level	<3 mg/dL	<3 mg/dL
	High sensitivity troponin	25 pg/mL	2-20 pg/mL
	3-hour high-sensitivity troponin	23 pg/mL	2-20 pg/mL
	B-type natriuretic peptide	136 pg/mL	<100 pg/mL
	Rapid plasma reagin	Negative	Negative
	Sedimentation rate	100 mm/h	0-15 mm/h
	C-reactive protein	142 mg/L	<5 mg/L
	Procalcitonin	0.28 ng/mL	<0.5 ng/mL
Hepatitis panel	Hepatitis A antibody, immunoglobulin M	Negative	Negative
	Hepatitis B surface antigen screen	Negative	Negative
	Hepatitis B core antibody, immunoglobulin M	Negative	Negative
	Hepatitis C antibody	<0.9 s/co ratio	0.0-0.9 s/co ratio
	Human immunodeficiency virus screen 4th generation with reflex	Non-reactive	Non-reactive
Urine drug screen	Benzodiazepine	Positive	Negative, none detected
	Cocaine	Positive	Negative, none detected
	Tetrahydrocannabinol	Negative, none detected	Negative, none detected
	Phencyclidine	Negative, none detected	Negative, none detected
	Amphetamine	Negative, none detected	Negative, none detected
	Opiate	Negative, none detected	Negative, none detected
	Methadone	Positive	Negative, none detected
	Blood culture 1	Candida albicans	Negative, no growth
	Blood culture 2	Candida albicans	Negative, no growth
	*Candida albicans* by PCR	Detected	Not detected
Antifungal AST 9 drug panel	Amphotericin B MIC	1.0 ug/mL	-
	Anidulafungin MIC	0.06 ug/mL susceptible	-
	Caspofungin MIC	0.06 ug/mL susceptible	-
	Fluconazole MIC	0.5 ug/mL susceptible	-
	Flucytosine MIC	0.12 ug/mL	-
	Itraconazole MIC	0.12 ug/mL	-
	Micafungin MIC	0.016 ug/mL susceptible	-
	Posaconazole MIC	0.06 ug/mL	-
	Voriconazole MIC	0.03 ug/mL susceptible	-

**Video 1:** Transthoracic echocardiogram focused on the mitral valve showing thickening of the posterior leaflet of the mitral valve.

**Video 2:** Transthoracic echocardiogram of the mitral valve showing trace mitral valve regurgitation.

**Video 3:** Transesophageal echocardiogram of the mitral valve showing mobile mass on the posterior leaflet measuring approximately 8.5×4.5 mm.

**Video 4:** Transesophageal echocardiogram showing mild mitral valve regurgitation.

## Discussion

A severe opportunistic infection, infective fungal endocarditis has a mortality rate of about 50% [5]. Similar to bacterial infections, the clinical manifestations may include fever, major peripheral embolization on echocardiography, neurological symptoms, dyspnea, chest pain, heart murmurs that change or appear, heart failure, and skin petechiae [5]. A growing number of patients with infective endocarditis suffer from fungal endocarditis, and fungal species make up 1-10% of all etiologic agents. Fungal endocarditis caused by *Candida albicans* accounts for 24-46% of all cases and prosthetic valve endocarditis for 3.4% [5].

It can be difficult to diagnose fungal endocarditis early because it often lacks the classic signs and symptoms of bacterial endocarditis [6]. In addition, it is challenging to prove that fungal endocarditis meets the modified Duke criteria since blood cultures are often negative, despite evidence of vegetations on echocardiograms [6]. Due to the non-specific presenting symptoms, a broad laboratory workup is often recommended in the acute setting [7]. A complete blood count often reveals a leukocytosis [3,7]. In around 60% of cases, inflammatory markers such as erythrocyte sedimentation rate (ESR) and c-reactive protein (CRP) are elevated [3,7]. A timely diagnosis and appropriate antimicrobial therapy are essential to reduce morbidity and mortality associated with fungal endocarditis. Due to the poor yield from blood cultures, which are positive fewer than 50% of the time, diagnosing fungal endocarditis is challenging [4]. Testing Mannan antigen, which is a cell wall component for Candida species, and antibody has a sensitivity and specificity of 83% and 86%, respectively [8]. Laboratory testing of 1,3 b-D-glucan, which is another fungal cell wall polymer, has a sensitivity and specificity of 69.9% and 87.1%, respectively [9]. Similar to the above, identifying galactomannan and 1,3 b-D-glucan can aid in identifying fungal endocarditis brought on by Aspergillus species [10].

Echocardiography is useful in the diagnosis and management of endocarditis [11]. Transthoracic echocardiography is quick, inexpensive, and non-invasive, with a 98% specificity for vegetations and a 60% overall sensitivity for vegetations [11]. When endocarditis or its consequences are highly suspected (for example, individuals with prosthetic valves, community-acquired staphylococcal bacteremia, or new atrioventricular block), even adequate TTE will not rule out endocarditis [11]. The next step would be to obtain a transesophageal echocardiogram (TEE) to better assess the valves of the heart. Higher ultrasonic frequencies increase spatial resolution and eliminate interference from intervening tissues in TEE pictures [11]. Because the TEE transducer in the esophagus is physically close to the aortic root and basal septum, where most such issues arise, TEE offers significantly better sensitivity (76-100%) and specificity (94%) [11]. TEE sensitivity can be increased further by imaging in two planes because incremental planes reduce the frequency of false-negative studies while also improving the characterization of vegetation area and mobility [11].

For the treatment of fungal endocarditis, an interdisciplinary approach is essential. In virtually all patients with fungal endocarditis, early valve replacement surgery (natural or prosthetic) of the diseased valve (class I indication) is suggested, as is a long course of antifungal therapy [4]. The first line of defense against Candida spp. endocarditis should be a lipid formulation of amphotericin B with or without flucytosine or a high-dose echinocandin (caspofungin or micafungin or anidulafungin) [4]. Step-down treatment with oral fluconazole (if susceptible) is advised once the patient has stabilized and follow-up blood cultures are negative. If the Candida isolate is resistant to fluconazole, oral voriconazole or posaconazole might be used [4]. The Infectious Diseases Society of America Candidiasis and American Heart Association Endocarditis guidelines recommend treatment of Candida endocarditis with either amphotericin B with or without flucytosine, or high-dose echinocandin therapy, followed by an oral azole for lifelong suppression [12].

## Conclusions

Here we presented an interesting case, to our knowledge, which has never been reported before, where a patient used a kitchen appliance to cause superficial dermal abrasions to increase the rate of his transdermal fentanyl patch absorption. He was ultimately diagnosed with fungal endocarditis and needed lifelong therapy. This study serves as an example of the value of a thorough and in-depth physical examination and history. The diagnosis of IE is not always simple, particularly when confounding circumstances are present, as they were in this study. This is an interesting and unusual presentation of fungal endocarditis.
